# Assembling an arsenal, the scorpion way

**DOI:** 10.1186/1471-2148-8-333

**Published:** 2008-12-16

**Authors:** Adi Kozminsky-Atias, Adi Bar-Shalom, Dan Mishmar, Noam Zilberberg

**Affiliations:** 1 Department of Life Sciences, Ben-Gurion University of the Negev, Beer-Sheva 84105, Israel; 2 Zlotowski Center for Neuroscience, Ben-Gurion University of the Negev, Beer-Sheva 84105, Israel

## Abstract

**Background:**

For survival, scorpions depend on a wide array of short neurotoxic polypeptides. The venoms of scorpions from the most studied group, the Buthida, are a rich source of small, 23–78 amino acid-long peptides, well packed by either three or four disulfide bridges that affect ion channel function in excitable and non-excitable cells.

**Results:**

In this work, by constructing a toxin transcripts data set from the venom gland of the scorpion *Buthus occitanus israelis*, we were able to follow the evolutionary path leading to mature toxin diversification and suggest a mechanism for leader peptide hyper-conservation. Toxins from each family were more closely related to one another than to toxins from other species, implying that fixation of duplicated genes followed speciation, suggesting early gene conversion events. Upon fixation, the mature toxin-coding domain was subjected to diversifying selection resulting in a significantly higher substitution rate that can be explained solely by diversifying selection. In contrast to the mature peptide, the leader peptide sequence was hyper-conserved and characterized by an atypical sub-neutral synonymous substitution rate. We interpret this as resulting from purifying selection acting on both the peptide and, as reported here for the first time, the DNA sequence, to create a toxin family-specific codon bias.

**Conclusion:**

We thus propose that scorpion toxin genes were shaped by selective forces acting at three levels, namely (1) diversifying the mature toxin, (2) conserving the leader peptide amino acid sequence and intriguingly, (3) conserving the leader DNA sequences.

## Background

The order *Scorpiones *constitutes one of the most ancient groups of animals on earth (more than 400 million years of evolution), represented today by approximately 1500 different species [1,2] which can be divided into four groups. The venoms of scorpions from the most studied group, the *Buthida*, are a rich source of small, 23–78 amino acid-long peptides, well packed by either three or four disulfide bridges, that affect ion channel function in excitable and non-excitable cells [1,2]. Typically, toxin peptides contain a structural signature defined by the presence of a cysteine-stabilized α/β motif, in which two disulfide bridges covalently link a α-helical segment with one strand of a β-sheet structure [3-5]. The dense core typically contains the sequence motifs, CXXXC and CXC (where C stands for cysteine and X stands for any amino acid residue), shown to be essentially conserved [1,2,6]. Toxins are mainly neurotoxic peptides, interacting specifically with various ionic channels (*i.e. *Na^+^, K^+^, Cl^- ^and Ca^2+ ^channels). The Na^+ ^channel toxins are gating modifiers that can be divided into two families, according to their pharmacological effect: α- and β-toxins. α-toxins bind in a voltage dependent mode and slow channel inactivation [7,8]. β-toxins bind to a separate site, independently of membrane potential, and shift the channel activation to the hyperpolarizing direction [7,8]. β-toxins can be further divided, according to their amino acid sequence and selectivity, into "true" β, excitatory and depressant toxins. As opposed to all others, the excitatory and depressant toxins are not toxic to mammals. Short chain toxins form a large family of peptides that block K^+ ^or Cl^- ^channels [5,9].

Within each toxin family, the amino acid sequence of the leader peptide is conserved, while the mature toxin-coding domain displays hyper-diversification, especially in those residues exposed to the surface of the molecule and which affect toxin activity [2,10]. These characteristics (*i.e. *conserved cysteine scaffold, conserved leader peptide and hyper-variable mature toxin) are shared with toxic peptides from other animal orders, such as snakes and cone snails [6,11,12]. In all these animals, evolution shows an apparent preference for toxin-diversifying processes [11,13].

Scorpions feed on insects, spiders and other small invertebrates. Consequently, scorpion venom contains toxins which effectively paralyze this type of prey. The venom, however, also serves a defensive function, and is, therefore, highly lethal to vertebrates, including mammals. This has been suggested as the driving force for the apparently accelerated rate of evolution displayed by mature toxins [14]. Although a number of studies have considered conopeptides and snake toxins from an evolutionary perspective [11,13], thus far, the mechanisms leading to accelerated evolution of scorpion toxins have not been thoroughly addressed.

Accordingly, to study the evolution of scorpion toxin gene families, in the present report we have unraveled the venom gland content of the Israeli scorpion, *Buthus occitanus israelis *(Boi). In all identified toxin families, the leader peptide sequences displayed remarkable conservation, owing to purifying selection forces aimed at conserving both the amino acid and nucleotide sequences, while the mature toxin-coding sequences have undergone diversifying selection. These observations provided the unique opportunity to study the evolutionary fate of gene families simultaneously undergoing opposite selective pressures in separate domains. The data obtained provide insight into the evolutionary pathways leading to the diversification of the mature toxin and imply the existence of a novel mechanism leading to family-specific leader peptide hyper-conservation.

## Results

### The venom gland contains a vast variety of neurotoxins

**Figure 1:**
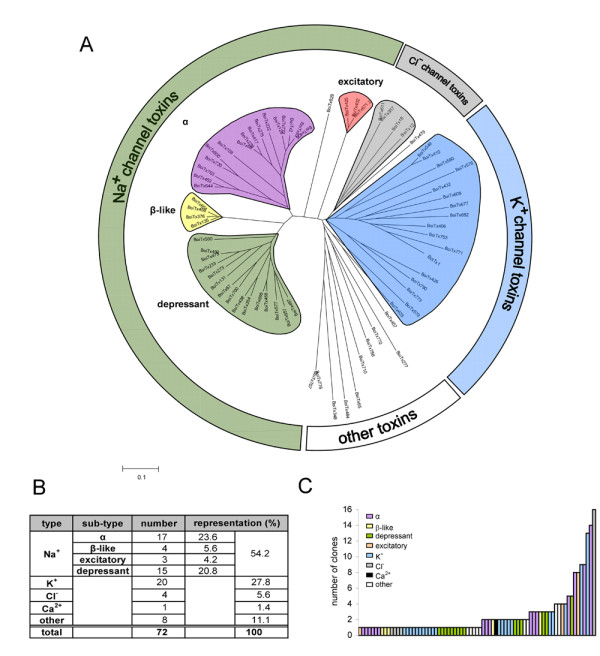
***B. occitanus israelis *venom gland transcript distribution**. (A) A phylogenetic tree analysis of putative toxins. Toxin families are highlighted according to their estimated blocking targets, *i.e. *Na^+^, K^+^, Cl^- ^or Ca^2+ ^channels, based on homology to known toxins [16]. Putative toxins with no known homologs were termed 'other toxins'. Only full-length distinct cDNA sequences were used for the protein data set construction. (B) Toxin families' transcript distribution. The number of distinct members of each family is presented as is their relative representation within the cDNA library. (C) Transcript expression levels. The number of clones obtained for each of the individual transcripts in the cDNA library is indicated. The estimated affiliation of each transcript is shown by shading of the bars.

To study the evolutionary mechanisms underlying the diversity of scorpion toxins, a database of all major transcripts expressed in the venom gland of the Israeli scorpion, *B. occitanus israelis *(Boi), was constructed. In addition, a venom-gland based cDNA library was established. To increase the chances of identifying rare toxin cDNA (*i.e. *frequency < 0.005), approximately 450 clones were isolated and sequenced. To identify putative toxin-encoding clones, translated cDNA sequences were first compared to those of known toxins. Next, all other open reading frames (ORFs) were inspected for the presence of a putative leader sequence or a known cysteine-based pattern. This sequencing effort showed 78% of the clones to have sequence attributes of toxins, leading these sequences to be termed putative scorpion toxin cDNAs. Of these, seventy two novel individual toxin-like transcripts were identified (GenBank accession numbers FJ360768–FJ360843). Evolutionary relationships between the toxins cDNAs were examined by the construction of a phylogenetic tree (Fig. 1A) based on alignments of both cDNA and predicted protein sequences. The Na^+^-channel toxin family and some of the K^+^-channel toxins could be clearly dividable into distinct sub-families on the basis of conserved elements present in the precursor sequences. Since the cDNA and protein trees presented highly similar topologies, only the protein tree is shown (Fig. 1A). As shown in figure 1B, over 50% of Boi toxin sequences are predicted to be Na^+ ^channel modifiers and can be divided, by sequence similarity, into 4 sub-families (Fig. 1B). The predicted K^+ ^channel blockers comprise 28% of the total transcripts and can be divided into numerous sub-types (Fig. 1B). This diverse toxin family is characterized by its star-shaped phylogenetic tree pattern (Fig. 1A). Four putative transcripts were predicted to belong to the chlorotoxin-like family. Eight additional ORF-containing putative transcripts, were identified as possessing toxin-like characteristics, *i.e. *a leader sequence and multiple cysteine residues, yet showed no similarity to known toxins. All Boi toxin precursors possessed the known two-domain structure, corresponding to the leader and mature peptide regions [5,15,16].

Striking differences in the transcription levels of different toxins could also be observed (Fig. 1C). Order-of-magnitude differences were observed between the relative expression levels of different transcripts from the same family, such as α-toxins, excitatory toxins as well as Cl^-^- and K^+^-channel toxins. Seventy five percent of the transcripts had relatively low transcript representation (≤ 2 independently isolated clones). Whereas, on the other hand, 5% of the transcripts had relatively high transcript representation (≥ 10 clones). Hence, related scorpion toxins may be expressed at vastly different levels in the venom gland.

### Are similar cDNA transcripts distinct genes or allelic variants?

Since the cDNA library was constructed from the pooled RNA from 10 different individuals, it was imperative to assess whether the observed transcript variety constituted the presence of multiple genes or was an outcome of the sample containing a much smaller number of multi-allele genes. To this end, all homologous transcript pairs with a percent identity of greater than 90% were studied. These correspond to four homologous cDNA pairs, namely: BoiTx1 and BoiTx260, putative K^+ ^channel toxins exhibiting 97% identity; BoiTx492 and BoiTx475, putative depressant toxins exhibiting 92.8% identity; BoiTx611 and BoiTx357, putative Cl^- ^channel toxins exhibiting 99% identity and BoiTx651 and BoiTx458, putative β-like toxins exhibiting 94.4% identity. The homologous pairs were subjected to PCR amplification using primers designed to identify both tested sequences. The templates for these experiments were the genomic DNA of all 10 scorpions from whence the cDNA library was generated. Individual cDNA clones served as control. In addition, the homologous cDNA pairs were digested with restriction enzymes directed against a recognition site found on only one of the clones in each pair (*i.e. Nco*I, *Hin*dIII, and *Lwe*I for pairs 1, 2 and 3, 4, respectively). Analysis of the amplification and restriction digestion products on agarose gel revealed that gene pairs 2, 3 and 4 exhibited digestion of only one partner of the pair in all 10 scorpions. On the other hand, clone pair 1 (97% identity) exhibited a single digestion pattern (*i.e. *heterozygous) in some individuals or no digestion at all in others. This digestion pattern implies that the BoiTx1 and BoiTx260 transcripts (*i.e. *pair 1) represent allelic variants of the same transcript, while all other sequences most likely correspond to separate genes.

### Toxin divergence occurred after speciation

**Figure 2:**
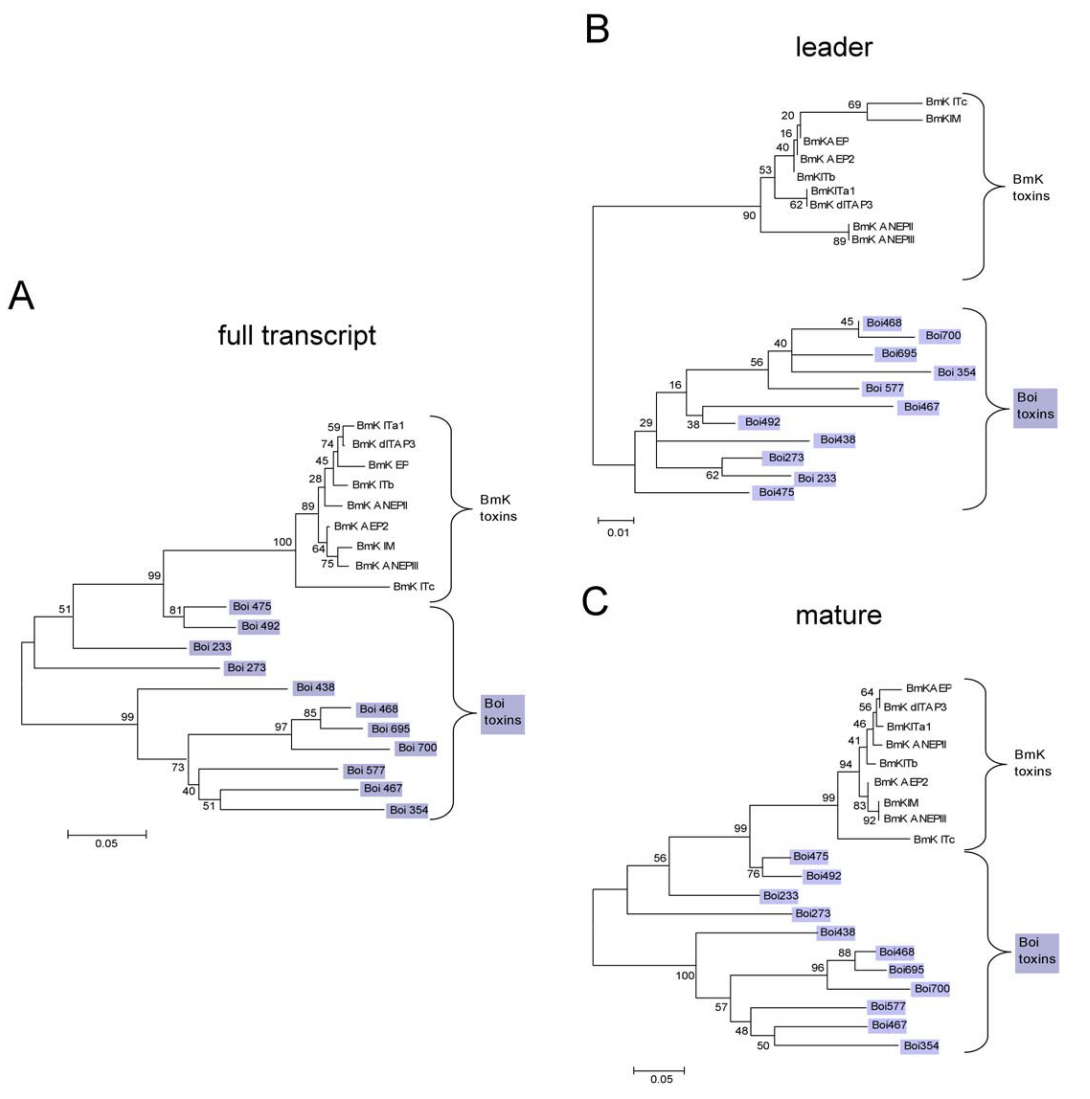
**Phylogenetic trees of the depressant toxin family of Boi and BmK scorpions**. (A) Full transcript, (B) leader-coding domain and (C) mature-coding domain of depressant toxins from both Boi and BmK scorpions were aligned by the MUSCLE algorithm and edited manually with Jalview. Trees were constructed by the neighbor-joining method, based on maximum likelihood composite. Numbers on the branches are bootstrap percentages, while brackets mark the genus origin of the toxins. Boi transcripts are shadowed in blue.

An attempt to decipher the relative time frame in which gene duplication events led to toxin divergence was next undertaken. At this point in time, a transcript dataset of the venom gland content from only a single scorpion species, namely *Buthus martensi *Karsch (BmK), gradually obtained over the course of the last few years [16], was available. cDNA sequences coding for the depressant toxin families from the two scorpions, *i.e*. Boi and BmK, were thus aligned and a neighbor-joining tree was constructed (Fig. 2A). Unexpectedly, most sequences in both families are more similar to others from the same species than to any of the sequences of the other species. Similar pattern was observed for the α-toxin family (not shown). If speciation was linked only to the evolution of the mature toxin one would expect the disappearance of the segregation when only the leader-coding sequence is considered. When examining separately the phylogenetic trees of the mature and the leader-coding domains (Fig. 2B, C), the leader-coding domain seemed to segregate even earlier than the mature toxin-coding domain. This will be further elucidated in the Discussion. Thus, these data can be interpreted as indicating that the two species diverged from a common progenitor before the duplication and diversification of toxins from the two families had occurred.

### Leader peptide conservation and mature toxin hyper-diversification

**Figure 3:**
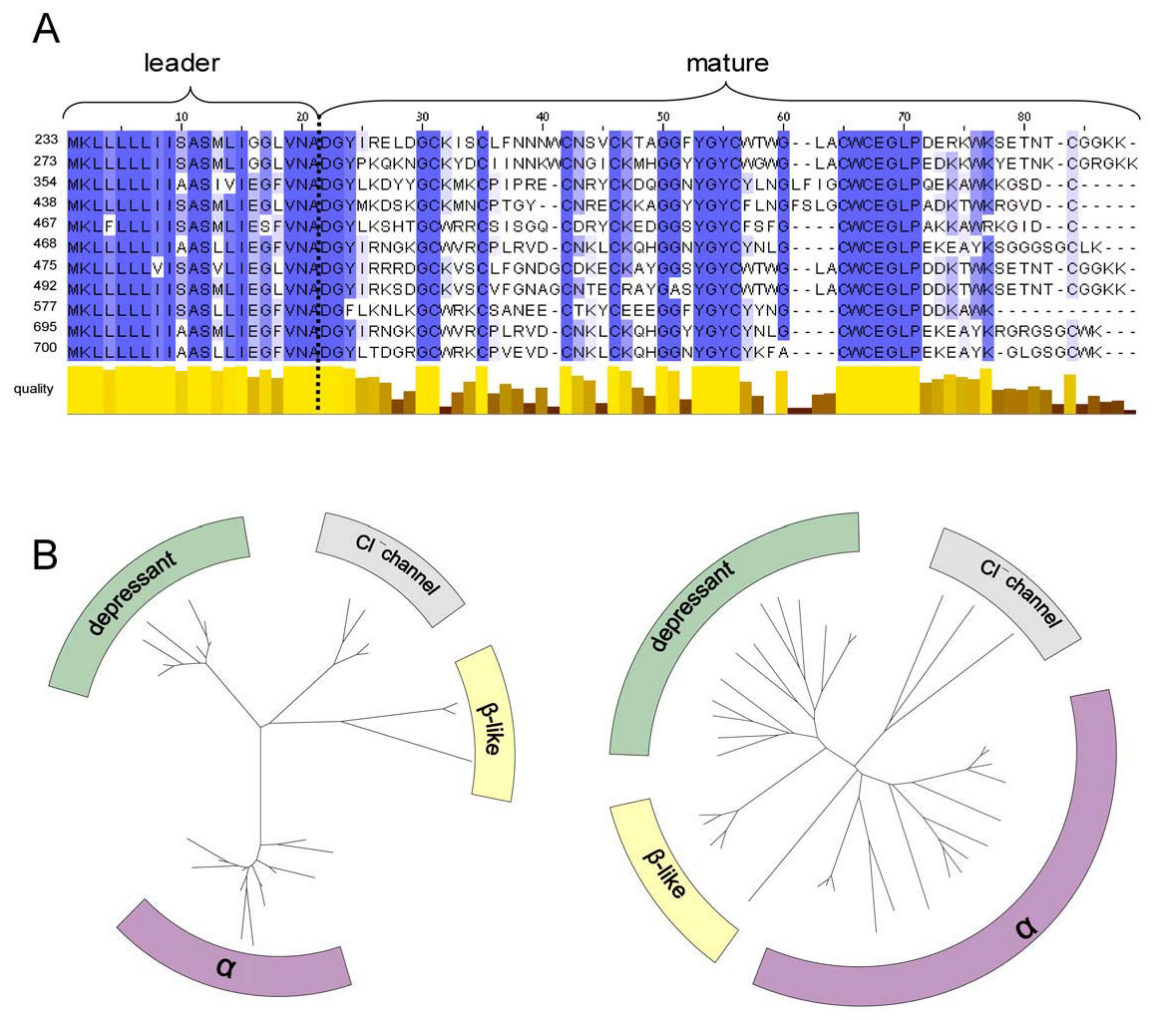
**Conservation levels within the coding region**. (A) Putative leader and mature domains of the depressant toxin family, as displayed by Jalview. Amino acid residues are shaded according to residue identity in a color scale from blue (high) to white (low). Position-specific similarities are indicated by the quality parameter (taking into account amino acid characteristics). The degree of similarity is indicated by bar height and color, ranging from light yellow (high) to dark brown (low). (B) Unrooted trees for the leader and the mature toxin regions of several toxin families. The toxin families' identity is indicated over each cluster. While in the leader domain, all the gene family segregate into clearly defined branches, in the mature domain, the transcripts start to diverge from a point much closer to the origin, in a star-like shape.

A high diversification of the mature toxin amino acid sequence was observed in several toxin families from cone snail and snake species [11,17-20]. To assess whether this pattern also held true for Boi, the most distinct toxin family, *i.e. *the depressant toxins, was first examined. The entire pool of coded peptides was aligned and is displayed according to the degree of conservation, as determine by Jalview (Fig. 3A). Such sequence alignment revealed high conservation of the leader domain, as opposed to the hyper-diversification of the mature domain. As expected, some highly conserved regions, such as those containing cysteine residues and other structurally important residues, were found within the hyper-diverse mature domain.

To examine whether other toxin families (*e.g. *α-, β-like and Cl^- ^channel toxins) possess the same pattern of conservation versus hyper-diversification, an unrooted neighbor-joining tree diagram of the different domains was plotted (Fig. 3B). While segregation of the different gene families is clearly observed in the leader domain (Fig. 3B, left plot), most transcripts diverge directly from the origin of the mature domain tree (Fig. 3B, right plot). The tight cluster of the leader domain tree, versus the star-like shape of the mature domain tree, demonstrates that the same pattern of conserved leader and diverse mature peptide sequence exists in all of the distinctive toxin families.

These data thus demonstrate that scorpion toxins gene families can be defined primarily on the basis of their highly conserved leader domains, in combination with distinctly conserved cysteine patterns, as was previously suggested for other scorpion species [16].

### The toxin gene domains experience opposite selective patterns

**Figure 4:**
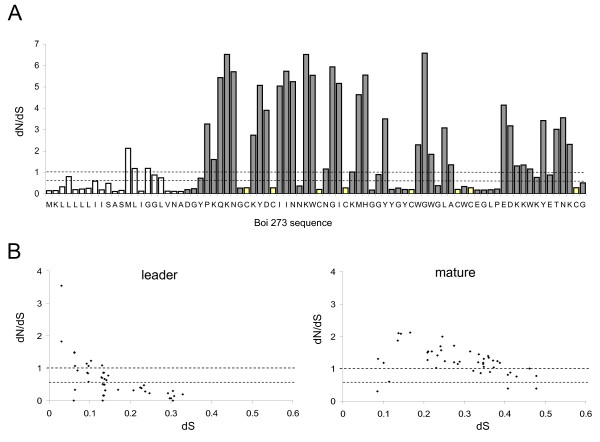
**Non-synonymous versus synonymous substitutions in the depressant toxin family**. dN over dS in intrafamilial comparisons of the leader and mature toxin coding domains. (A) Residue specific plot. Leader and mature residues are indicated by white and gray bars, respectively. Cysteine residues are indicated by yellow bars. (B) Matrix alignment plots were utilized to establish the correlation between the synonymous substitution rates and dN/dS ratios. Two lines distinguishes between values indicating positive (>1), neutral (0.6–1) and negative (<0.6) selections.

Positive selection has been suggested as playing a decisive role in the diversification of mature toxins in gene families of cone snails, snakes and scorpions [11,13,17,19,21-23]. Accordingly, it was hypothesized that in scorpions, selection could underlie both hyper-diversification of the mature toxin and conservation of the toxin leader domain. Therefore, the number of synonymous (dS) and non-synonymous (dN) substitutions per site in the toxin sequences, was assessed. Such analysis was performed on the two main toxin families identified within Boi venom gland, namely the α- and depressant toxin families. dN/dS values of 0.6 to 1.0 are indicative of neutral selection forces. Using the MEC model [24], the majority (55%) of the mature domain residues showed very strong positive selection, with 34% being under very strong negative selection and only 11% revealing a pattern interpreted as neutral selection. A similar degree of positive selection was found in both the Boi and BmK α-toxin families (not shown). Atypically, the degree of diversification within the BmK depressant toxin family was significantly low, leading to a small number of positively selected sites within the mature toxin sequence. This could be a result of a partial representation of the full variety of this specific toxin family in the databases and/or an indication of unusual evolutionary forces. Opposite to the mature toxin residues, the majority of the leader domain residues showed very strong (62%) negative selection, with only 5% being under the effect of positive selection forces and 33% being neutrally selected. The same pattern was obtained by the more promiscuous M5 and M8 models (*p *< 0.001) for both the depressant and α-toxin families (not shown). To establish the correlation between synonymous substitution rates and dN/dS ratios, DNAsp software was utilized. Again, the majority of the leader data points (Fig. 4B, left plot) argue in favor of a purifying selection (dN/dS < 0.6), while the majority of the mature domain data points (Fig. 4B, right plot) are indicative of diversifying selection (dN/dS>1). In addition, an apparent decrease in the synonymous mutation rate within the leader domain was observed, in comparison to the rate observed within the mature toxin (Fig. 4B) and will be discussed in the following sections.

### Apparent position-specific codon conservation in strong negatively-selected sites

**Figure 5:**
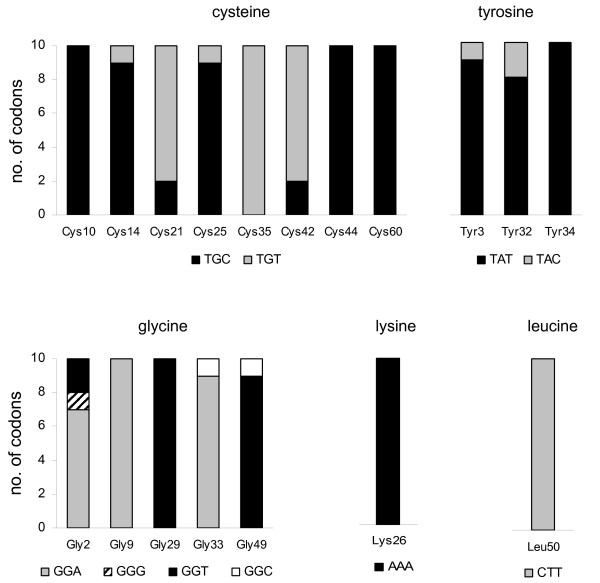
**Position-specific codon conservation in the depressant toxin family**. Codon usage of all negatively-selected sites within the mature toxin domain of ten depressant toxin family members is presented. Bars are shaded according to the codon representation, as indicated below. Amino acid numbers are according to BoiTx273 sequence.

Positions in the mature peptide that experience purifying selection not only show a strong conservation of the amino acid but also a position-specific codon conservation pattern. This phenomenon was observed within the depressant toxins family (Fig. 5), as well as in other families (not shown). Codon conservation appears not to be a consequence of codon usage bias as different codons are conserved at different positions. For example, for the cysteine at positions 3, 5 and 6, the conserved codon is TGT, while at all other cysteine-coding positions, the TGC codon is conserved (Fig. 5). In addition, analysis of the entire Boi transcript data set and assessment of codon abundance shows that the conserved codon is often not the most abundant codon in the transcript library.

### Nucleotide diversity is greater within the mature toxin-coding domain than in other gene domains

**Figure 6:**
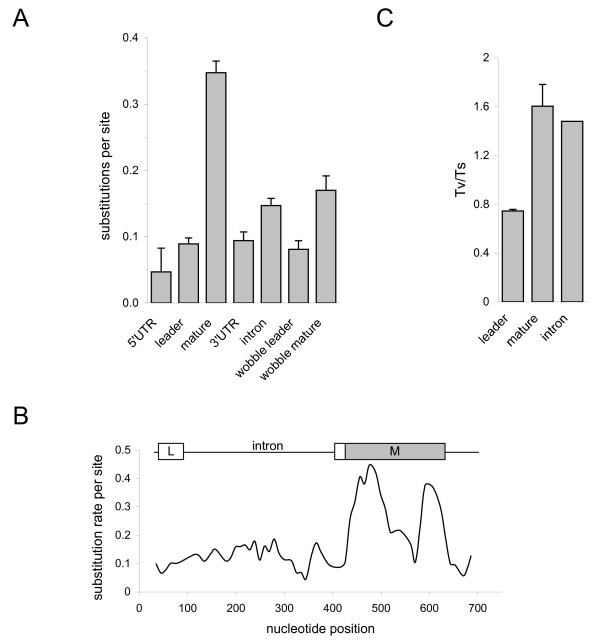
**Nucleotide diversity of the depressant toxin family gene domains**. (A) The nucleotide diversity (presented as the number of substitutions per site) of the depressant toxin family gene domains, as well as at the wobble position of the negatively-selected sites in the leader and mature-coding domain, was calculated by the Jukes and Cantor parameter model [25]. The different gene domains are indicated. (B) Sliding window analysis of the substitution rate per site (pI values) across the depressant toxins gene family. A high substitution rate was found in the mature toxin coding region (M), as compared with the gene regions coding for the leader (L), the intron or the untranslated regions (unmarked), as indicated above. (C) The transversions to transitions (Tv/Ts) ratio in Boi genes. Tv/Ts ratio was calculated for the leader and mature regions of the depressant and α-toxin families. Tv/Ts averages and SEM values for each region are shown. For comparison, the Tv/Ts ratio of the depressant toxin family introns is presented.

Selective forces are expected to impose a higher substitution rate per site within the mature-coding domain than within the leader sequence. Indeed, when analyzing the depressant toxin gene family by the Jukes and Cantor parameter model [25], more than 3-fold higher nucleotide diversity was observed within the mature-coding domain than in the other coding and non-coding domains (Fig. 6A). This pattern was repeated when analyzing the depressant toxin gene family from BmK and the α-toxins gene family from Boi.

Can this difference in substitution rates between the leader and the mature domains be attributed solely to the influence of selective forces? To assess this possibility, the nucleotide sequences of all Boi depressant toxin introns were isolated and determined. One of the depressant toxins genes lacked an intron. All other genes possess a 298 to 327 bp-long intron, located near the 3' end of the leader-coding sequence, at a position similar to other scorpion toxins genes [26,27]. This intron domain is thought to reflect a neutral nucleotide mutation-fixation rate. Indeed, Boi toxin intron nucleotide diversity exceeds that of the leader-coding domain but is much lower than that of the mature toxin coding domain (Fig. 6A, B). To assess the neutral substitution rates affecting the coding domains while eliminating the effects of protein-shaping selective forces, the substitution rates per site in the wobble (3^rd ^codon) position of the negatively-selected sites were determined (Fig. 6A). The neutral substitution rate within the mature toxin coding region was similar to that of the intron. On the other hand, the neutral substitution rate within the leader-coding region was significantly (~2-fold) lower (Fig. 6A). This can be interpreted as an indication of a decrease in the synonymous substitution rate and of position-specific codon bias in the leader-coding domain.

### Transition preference in the leader toxin coding domain

We further characterized Boi's toxin genes dataset by examining the transversion/transition (Tv/Ts) ratios in depressant and α-toxins. We found a two-fold excess of transitions in the leader sequence in comparison with that of the mature-coding domain (Fig. 6C). To ascertain the neutrally selected Tv/Ts ratio, we examine this ratio within the intron region. The Tv/Ts ratio of the mature-coding domain was very similar to that of the intron while the leader was found to be under a strong transition bias.

## Discussion

Scorpion venoms have evolved during the last 400 million years to constitute an arsenal of high affinity ion channel modulators. Here, we investigated evolutionary pathways leading to the establishment of this pharmacological wealth by constructing and analyzing a Boi toxin cDNA library. The cDNA library transcripts exemplify the tremendous variety of toxin genes prevalent in an old world scorpion. The apparent hyper-diversity within each toxin family could be explained, in part, by the presence of multiple alleles per gene. Our comparison of genomic DNA versus cDNA sequences in several scorpion individuals indicates that in all but one of the cDNA clone pairs, diversity is not likely to reflect allelic variants but rather the existence of distinct genes. The degree of diversification within the different toxin families was versatile. Boi putative K^+ ^channel blockers are much more diverse than Na^+ ^channel modifiers, within the same activity sub-type (*i.e*. depressant, excitatory, α- and β-toxins) (Fig. 1A). We thus speculate that the high divergence of K^+ ^channel toxins is due to the higher variance in possible targets (*e.g*. mammals have ~10-fold more K^+ ^channel sub-types than Na^+ ^channels).

### Gene duplications upon speciation

The number of toxin genes in a single scorpion species indicates that several gene duplications occurred during scorpion evolution. Gene duplication is typically a consequence of unequal crossing-over, retro-positioning or chromosomal duplication [28]. In another species, the sea anemone *Nematostella vectensis*, it was recently suggested that toxin gene duplication was achieved by unequal crossing-over [29]. Although most scorpion toxin genes could have arisen via gene duplication, we have identified here, for the first time, a Boi depressant gene lacking the conserved intron located within the leader sequence coding domain. This molecular feature is indicative of the retro-positioning which occurs when an mRNA is retro-transcribed to cDNA and then inserted into the genome.

Following examination of toxin diversity in several venomous animals (snakes and cone snails [11,17,18,20]) it was suggested that duplication of the toxin genes followed speciation. Our inter-species examination of depressant and α-toxin families in Boi and BmK scorpions revealed that most of the toxin subfamilies are unique to each species, suggesting again that speciation preceded gene duplication.

Here we suggest a possible evolutionary mechanism enabling fixation of toxin gene duplicates upon speciation. Similar to point mutations, duplications occur in an individual, which can be either fixed or lost in the population. If the new allele is selectively neutral, as compared with pre-existing alleles, that allele has only the small probability of 1/2 N of being fixed in a diploid population [30], where N is the effective population size. This suggests that the vast majority of the duplicated genes will be lost. The speciation process entails a vast decrease in the new effective population size, thus opening a short window of time enabling an increase in the fixation rate of gene duplication. Therefore, the association of duplication with speciation could be due to a rise in the duplication fixation rate upon speciation, caused by a decrease in the effective population size. For those duplicated genes that do become fixed, fixation is time-consuming. On average, it takes 4 N generations for a neutral allele to become fixed in a population of N individuals [30]. Furthermore, the number of toxins in each family is similar in the two scorpions, as is the overall number of toxins in the venom. The latter was shown to also be the case in other scorpions [31], as well as with other species [11,32]. Such observations imply that upon a dramatic change in the environment that promotes speciation, rapid fixation rate of duplicated toxin genes occurs so as to allow the introduction of novel toxic phenotypes. At the same time, a similar number of toxin genes are lost. This may allow for adaptation to novel ecological niches and pray, while maintaining a minimal concentration of each of the toxins within the limited venom pool, as was recently suggested for the PLA_2 _genes of snakes [23].

Could gene conversion cause the intra-species toxin similarity? In the sea anemone *N. vectensis *[29], as well as in two species of sea snakes [33,34], a very high degree of conservation has been reported within certain toxin families, suggesting the involvement of concerted evolution processes [29]. Here, the degree of diversity among paralogous genes roles out the possibility of recent gene conversion, although the higher degree of similarity between paralogous genes, as compared with orthologous genes, can not rule out the occurrence of early gene conversion events [35].

### Mature toxin gene diversification

Protein neofunctionalism after gene duplication is believed to have played a major role in evolution [36], although the mechanisms by which this process occurs remains controversial. When comparing the amino acid sequences of protoxins from all families, hyper-diversification of the mature toxin and conservation of the leader peptide were observed (Fig. 3). In this study, we examined the Boi depressant toxin gene family to evaluate the evolutionary processes leading to this phenomenon. Within the mature-coding region, diversifying forces (Fig. 4) are sufficient to explain the high observed substitution rate (Fig. 6A, B) given that the neutral substitution rate within the synonymous sites (*i.e*. substitution rate at the wobble position at negatively-selected sites) is the same as that of the mostly neutral intron (Fig. 6A). The high substitution rate of the positively-selected sites gave rise to alleged position-specific codon conservation at adjacent negatively-selected sites (Fig. 5).

### Leader toxin hyper-conservation

**Figure 7:**
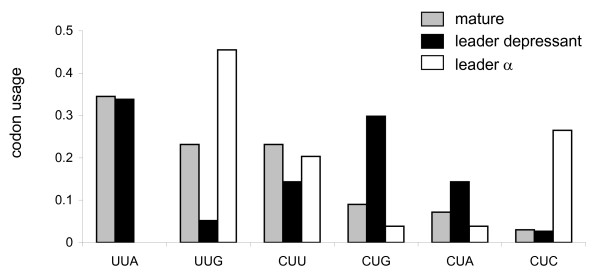
**Codon usage of leucine residues at the different gene domains**. The codon usage of leucine residues within the leader-coding regions of the depressant and α-toxin families is compared to that of the mature-coding domain of all toxins within the data set.

Toxin family-specific amino acid conservation within the leader peptide is apparent in the venoms of scorpions, cone snails [11,18], snakes and spiders. This is surprising as in most secreted proteins, only very basic biochemical characteristics of the leader peptide are maintained, with the amino acid sequence being variable [37]. We thus proceeded to elucidate a possible rationale for the amino acid conservation within the leader peptides of toxins. A strong negative selection was observed for most leader peptide residues within Boi toxin genes, specifically at the amino terminal (Fig. 4A). Is this the only force imposing strong nucleotide conservation in this gene domain? Examining the neutral substitution rate of the leader (within the wobble position at negatively selected sites) revealed a substantially lower rate than that of the neutral intron (Fig. 6A), implying the existence of purifying selection against synonymous sites. This is probably not the consequence of a recent exon-specific gene conversion, as certain residues within this region are relatively variable (Fig. 4A). We examined the codon usage within the leader of each of the two main toxin families (depressant and α-toxins) and identified a family-specific pattern. This pattern was unique to each family and different from that of the mature-coding domain of each family and of the combined data set of all Boi mature toxin-coding regions. This observation was statistically significant (chi square-test, *p *< 0.001). On the other hand, the codon usage of the mature toxin coding domain of each of the families was not significantly different than that of the entire Boi mature toxin coding regions data set. For example, the most frequently used codon for leucine, the most abundant amino acid within the leader, for α-toxin is UUG. By contrast, this codon is hardly used in the depressant toxin leader sequence (Fig. 7). The rarely used codons, CUG and CUC, are abundant within the depressant and α-toxin leader sequences, respectively (Fig. 7).

The strong inter-species leader conservation, as indicated by their defined segregation between Boi and BmK scorpions within depressant (Fig. 2B) and α-toxin families (not shown), supports our suggestion for a codon usage-dependant translational regulatory mechanism. This implies that a transcriptional regulatory mechanism may be at work that differently affects the toxin families tested and is species-specific (Fig. 2B), as expected. Although, we can not rule out other mechanisms for non-neutral evolution at synonymous sites, such as regulation of mRNA stability and/or intron splicing [38], here we propose that the expression levels of toxin families are differentially controlled via regulation at the translation level. Our suggested mechanism exploits the relative abundance of different tRNA species to either restrict or enhance translation rates.

In addition, a clear bias for transitions over transversions was observed in the leader-coding domain in comparison to the Tv/Ts ratio within the intron and the mature toxin-coding domain (Fig. 6C). This could be attributed to strong negative selection forces inflicted upon this region, as at the most variable site, *i.e.*, the wobble position, a transition would be preferred since in 94% of cases, this would result in a silent synonymous mutation.

In summary, positive selection upon mature toxin residues was reported in the venoms of other venomous organisms, such as snakes [12,13,39], cone snails [11,17] and spiders [32], suggesting a common diversifying evolutionary mechanism. Indeed, hyper-variability in gene families is apparent in systems evolved to recognize foreign molecules, whether those genes encode venom-derived toxins, gene families of the immune system or antigenic parasite surface proteins [40]. In cone snails, a hyper-variability-generating molecular mechanism relying upon an error prone-like DNA polymerase was suggested [11].

In this work, by constructing and analyzing a dataset of toxin transcripts from the venom gland of the scorpion Boi, we were able to follow the evolutionary path leading to scorpion toxin diversification. Duplicated genes encoding toxin peptides were fixed within the genome mainly following speciation. Upon such fixation, the mature toxin coding domain was subjected to diversifying selection to increase species fitness. This resulted in alleged position-specific codon conservation at adjacent negatively-selected sites. On the other hand, the leader peptide is characterized by a conservation of the amino acid sequence, a sub-neutral synonymous substitution rate and a strong transition bias. This was explained by purifying selection forces acting to conserve both the peptide and DNA sequences. This suggests the presence of a family-specific, codon-sensitive translational regulatory mechanism for toxin genes.

## Methods

### cDNA library construction and screening

*Buthus occitanus israelis *(Boi) scorpions were collected at Sde Boker, Israel. Total RNA was extracted from the venom glands of 10 scorpions using an EZ RNA extraction kit (Promega), 2 days after electrical 'milking' of the venom. mRNA was purified with a PolyATract mRNA Isolation System (Promega). Double-stranded cDNA was synthesized from 2 μg of mRNA using the Universal Riboclone cDNA Synthesis System (Promega). Blunt end cDNA clones were cloned into the pBluescript KS^+ ^(pBS) plasmid digested with *Sma*I and transfected into *Escherichia coli *DH5α cells. Electro-transformation of the cDNA ligation yielded a library comprising approximately 5 × 10^4 ^primary clones. To determine insert size, individual clones were amplified by PCR using T7 and T3 primers. To avoid sequencing through the poly A tail, insert orientation was determined by PCR using the T3, T7 and poly T primers. Four hundred and twenty randomly chosen cDNA clones, ranging in length from 250 to 600 bp, were then sequenced to obtain a reliable representation of the toxin content in the venom gland.

### Molecular biology

Plasmid DNA was purified using a Wizard plus SV Miniprep kit (Promega). Restriction enzyme digestions, DNA ligations and phosphorylation and dephosphorylation reactions were performed according to manufacturer instructions (Fermentas or New England Biolabs). Competent *E. coli *DH5α cells were transformed by either heat shock or electroporation procedures. Polymerase chain reactions (PCR) were performed with a PTC-2000 apparatus (MJ Research), using *Pfu *(Fermentas) or *Taq *(Promega) enzymes, as indicated.

### Differentiation between genes and alleles

Genomic DNA was isolated from the same 10 scorpions used for the cDNA library construction. Four pairs of homologous genes exhibiting over 90% identity were selected for analysis. As such, four PCR primer pairs were designed, according to the cDNA sequence, to amplify each gene pair. To distinguish between the clones, digestion by a restriction enzyme direct against a recognition site found on only one of the genes in each pair was preformed.

### Sequencing the introns of depressant toxins genes

Introns from the depressant toxin family were amplified using *Pfu *DNA polymerase with Boi genomic DNA as template. PCR forward primers were constructed to match a highly conserved sequence of the 5' UTR and leader regions. Reverse primers were designed to match a hyper-variable region within the mature toxin-coding domain to ensure specificity. PCR amplification products were cloned into a *Sma*I-digested pBS vector. Positive clones were then sequenced to retrieve the intron sequence.

### Evolutionary analysis

Toxin classification and homology identification was achieved using the BLASTN and BLASTP programs [41]. Individual transcripts were aligned using MUSCLE algorithm [42]. Alignments were refined manually by the Jalview program [43]. Unrooted phylogenetic trees were constructed using the neighbor-joining method and maximum likelihood algorithms [44] using MEGA4 software [45] and then visualized with TreeView [46]. Synonymous versus non-synonymous substitution rates were analyzed using the DNAsp [47] and Selecton [24] programs. Transitions versus transversion rates, as well as codon usage profiles, were drawn using MEGA4 software. Nucleotide diversity rates were estimated by the DNAsp program.

## Authors' contributions

AKA constructed the cDNA library, performed the molecular evolution analysis and drafted the manuscript. ABS helped in the cDNA library screening. DM participated in data analysis and manuscript revision. NZ conceived the study, and participated in its design and coordination and helped to draft the manuscript.
